# Supplementary material for: Optimizing Surface Characteristics of Stainless Steel (SUS) for Enhanced Adhesion in Heterojunction Bilayer SUS/Polyamide 66 Composites
Source: Polymers (Basel). 2024 Sep 27;16(19):2737. doi: 10.3390/polym16192737 (PMC11478409; doi:10.3390/polym16192737)
Supplement: Supplementary file 1 [file polymers-16-02737-s001.zip › polymers-3188365-supplementary.pdf]

Supplementary information

Optimizing Surface Characteristics of Stainless Steel (SUS) for Enhanced Adhesion in Heterojunction Bilayer SUS/Polyamide 66 Composites

Sang-Seok Yun [1,†], Wanjun Yoon [2,†] and Keon-Soo Jang [1,*]

[1] Department of Polymer Engineering, School of Chemical and Materials Engineering, The University of Suwon, Hwaseong-si 18323, Republic of Korea
[2] CS Innovation Co., Hwaseong-si 18559, Republic of Korea
[†] These authors contributed equally to this work.
[*] Correspondence: ksjang@suwon.ac.kr

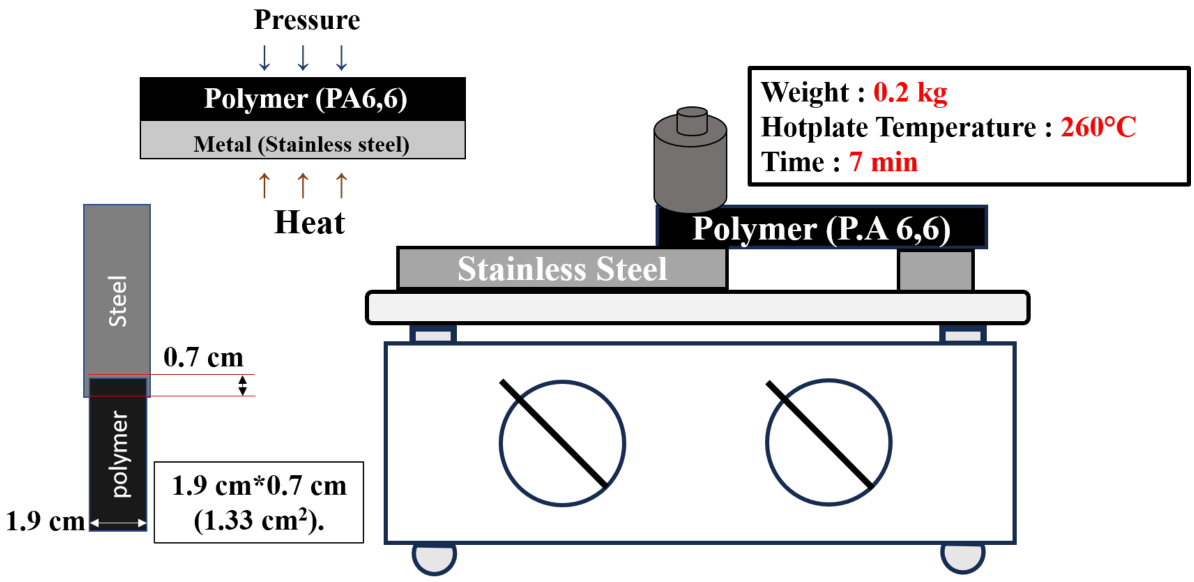

Figure S1. Bonding process for PA66/SUS bilayer composites.

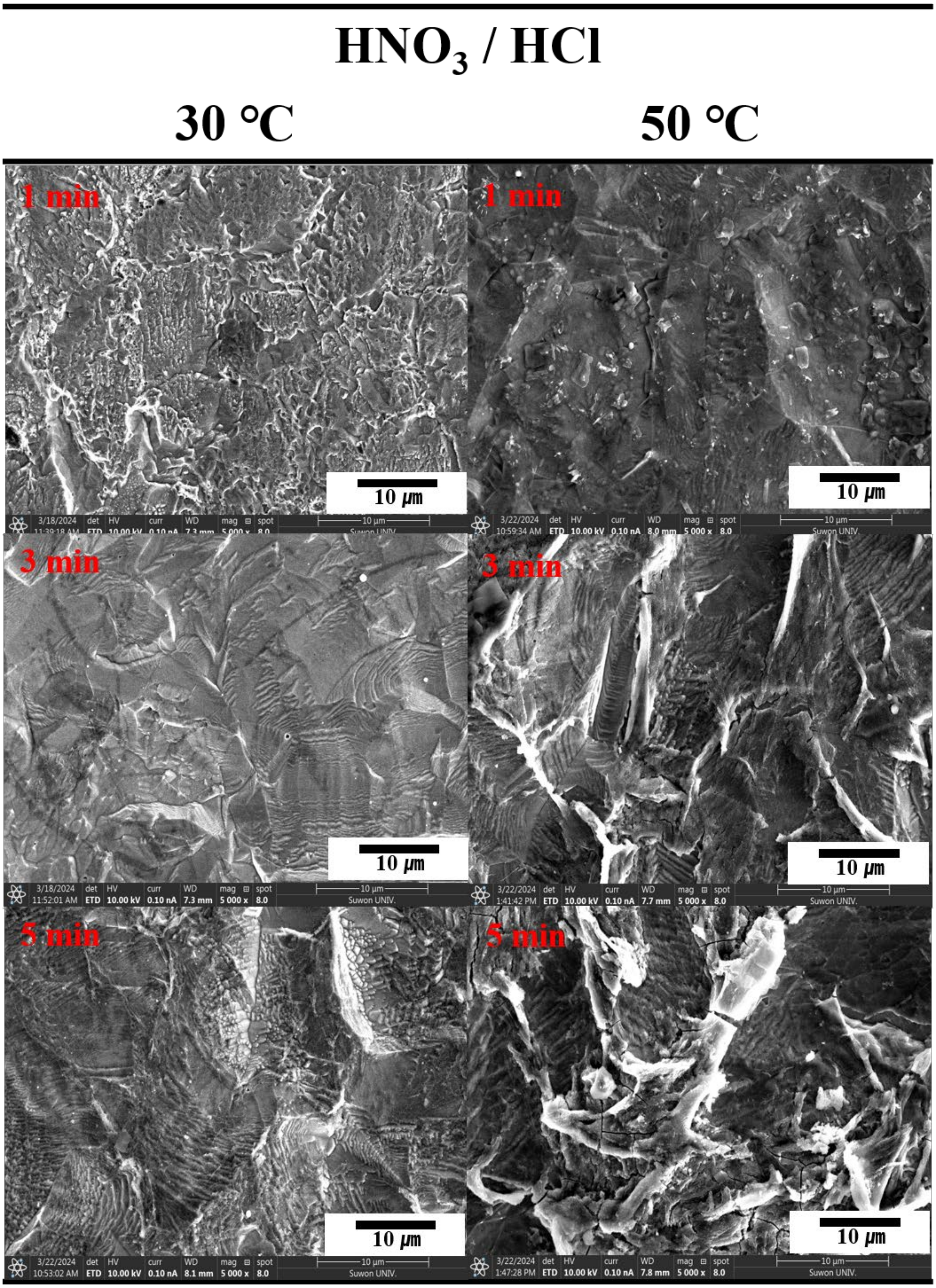

Figure S2. SEM images of SUS surfaces treated by HNO_3 + HCl with different etching temperatures and times before annealing.

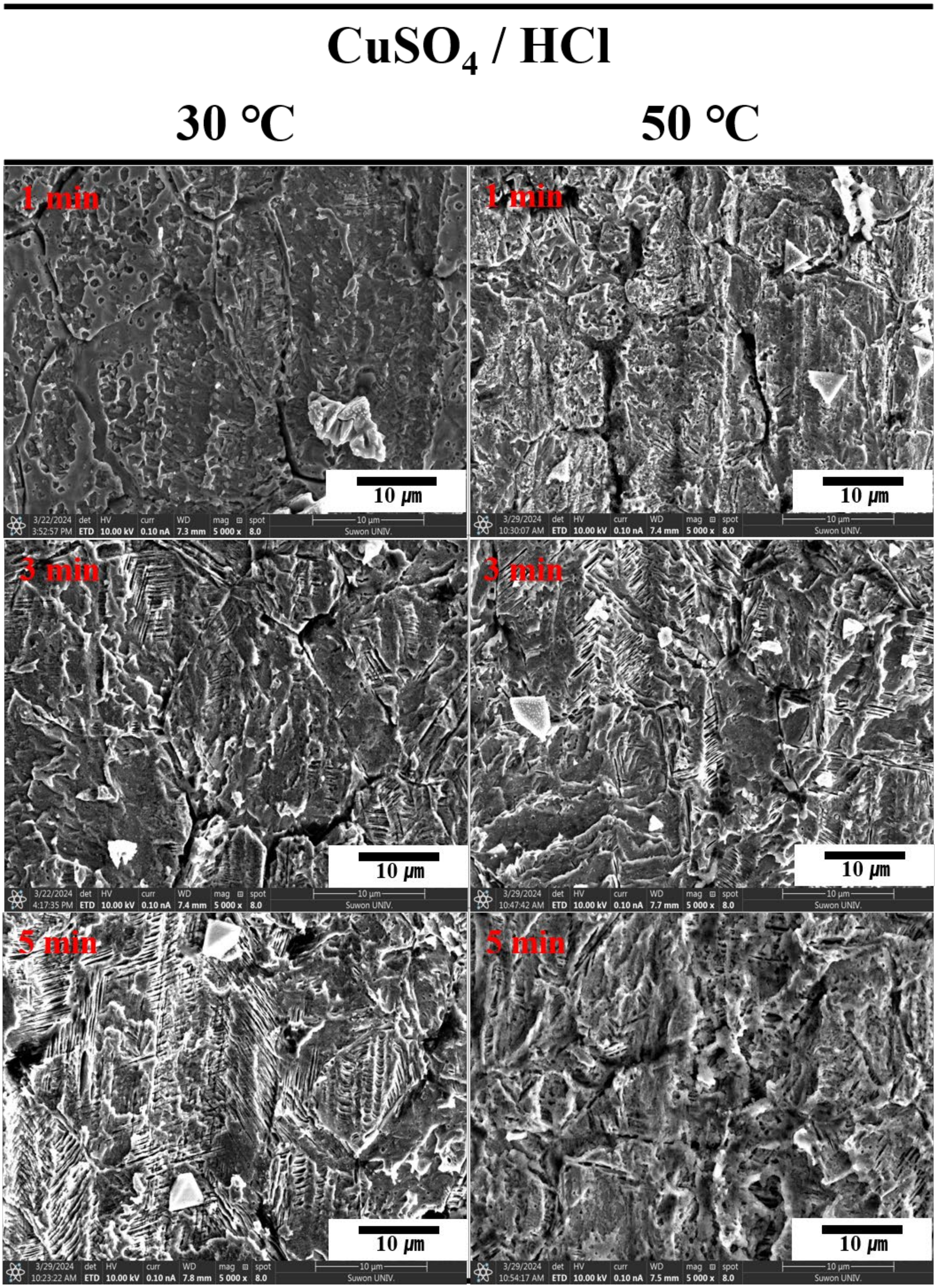

Figure S3. SEM images of SUS surfaces treated by $CuSO_4$/HCl with different etching temperatures and times before annealing.

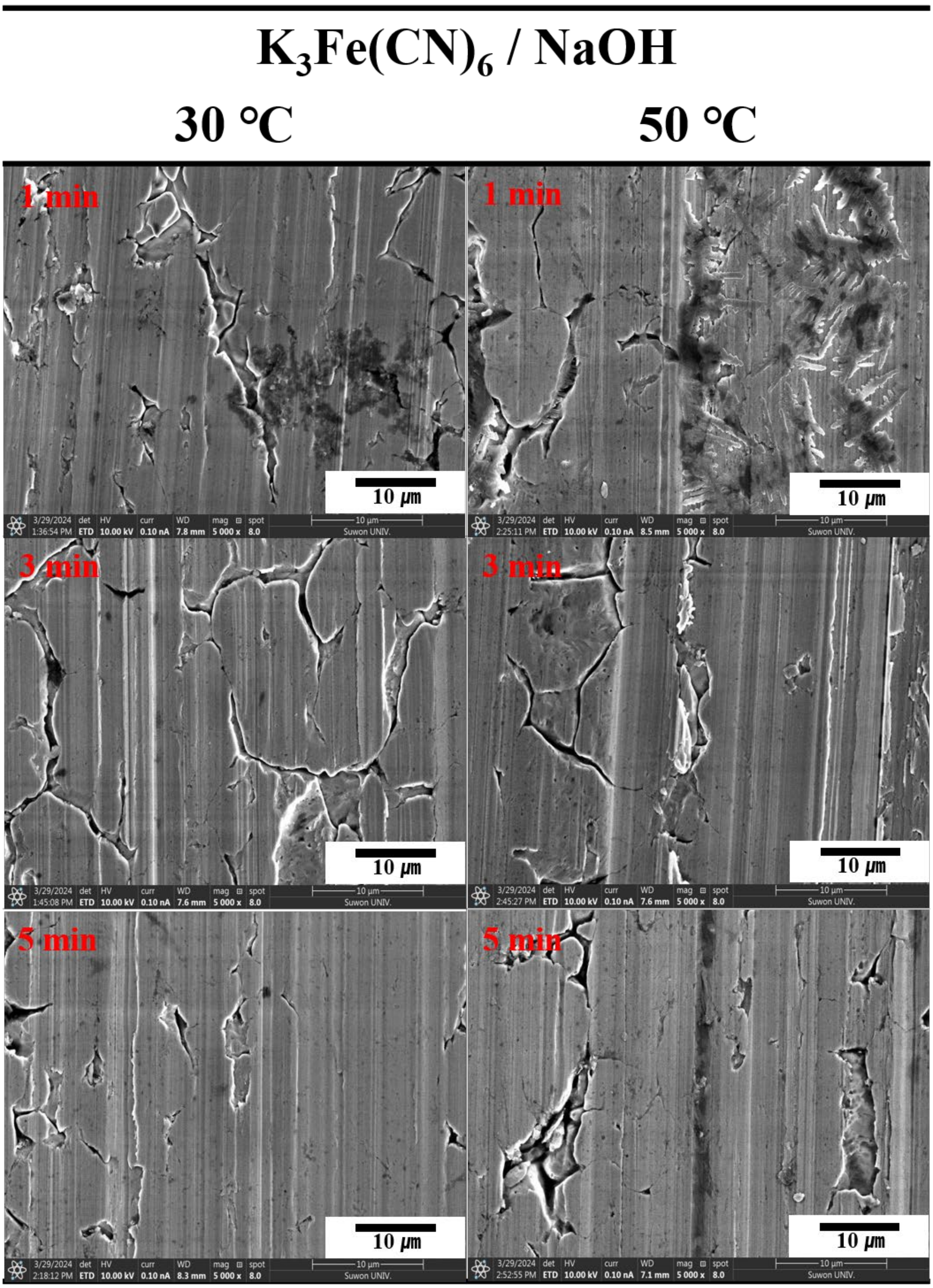

Figure S4. SEM images of SUS surfaces treated by $K_3Fe(CN)_6$/NaOH with different etching temperatures and times before annealing.

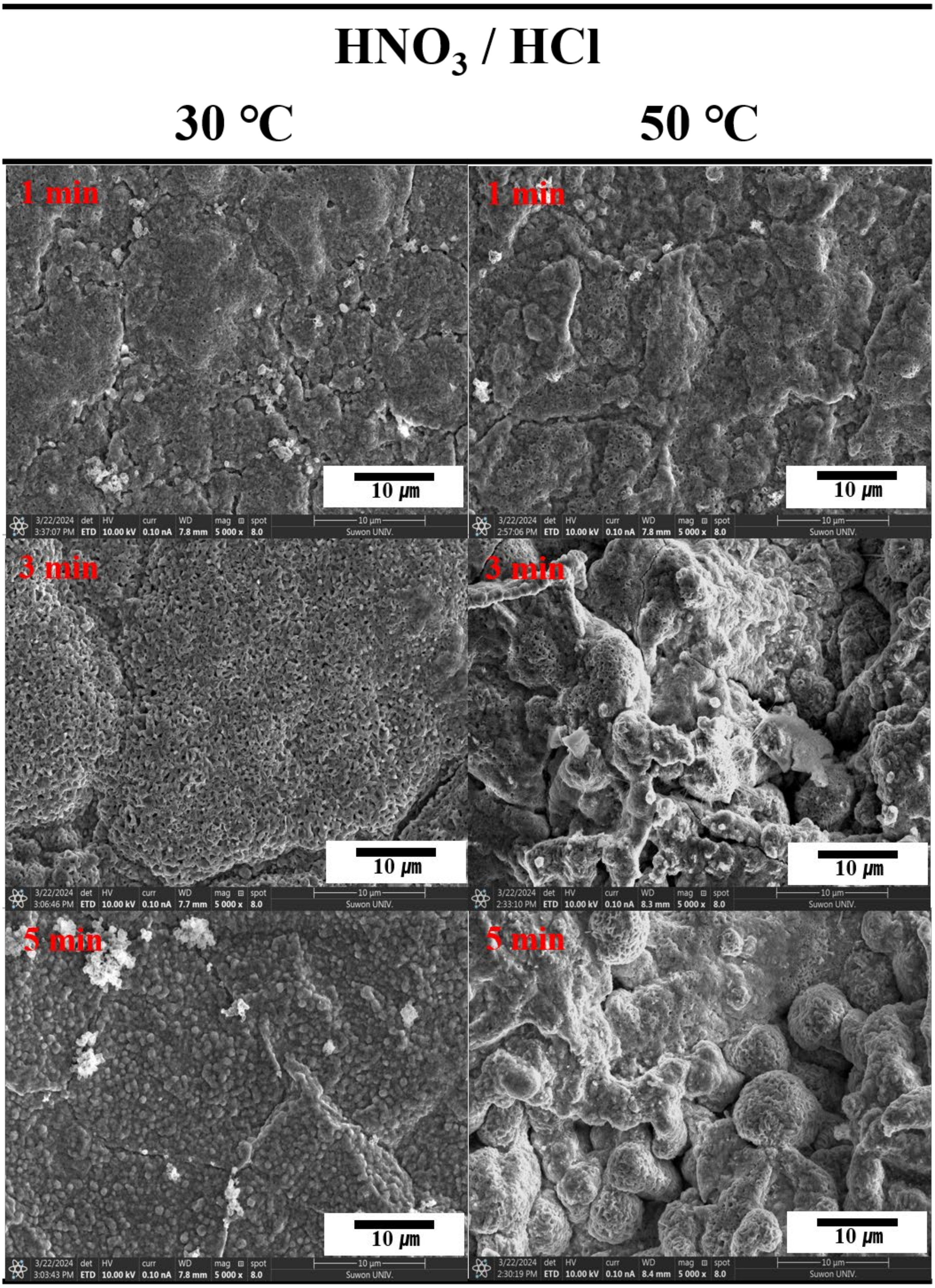

Figure S5. SEM images of SUS surfaces treated by HNO_3/HCl with different etching temperatures and times after annealing.

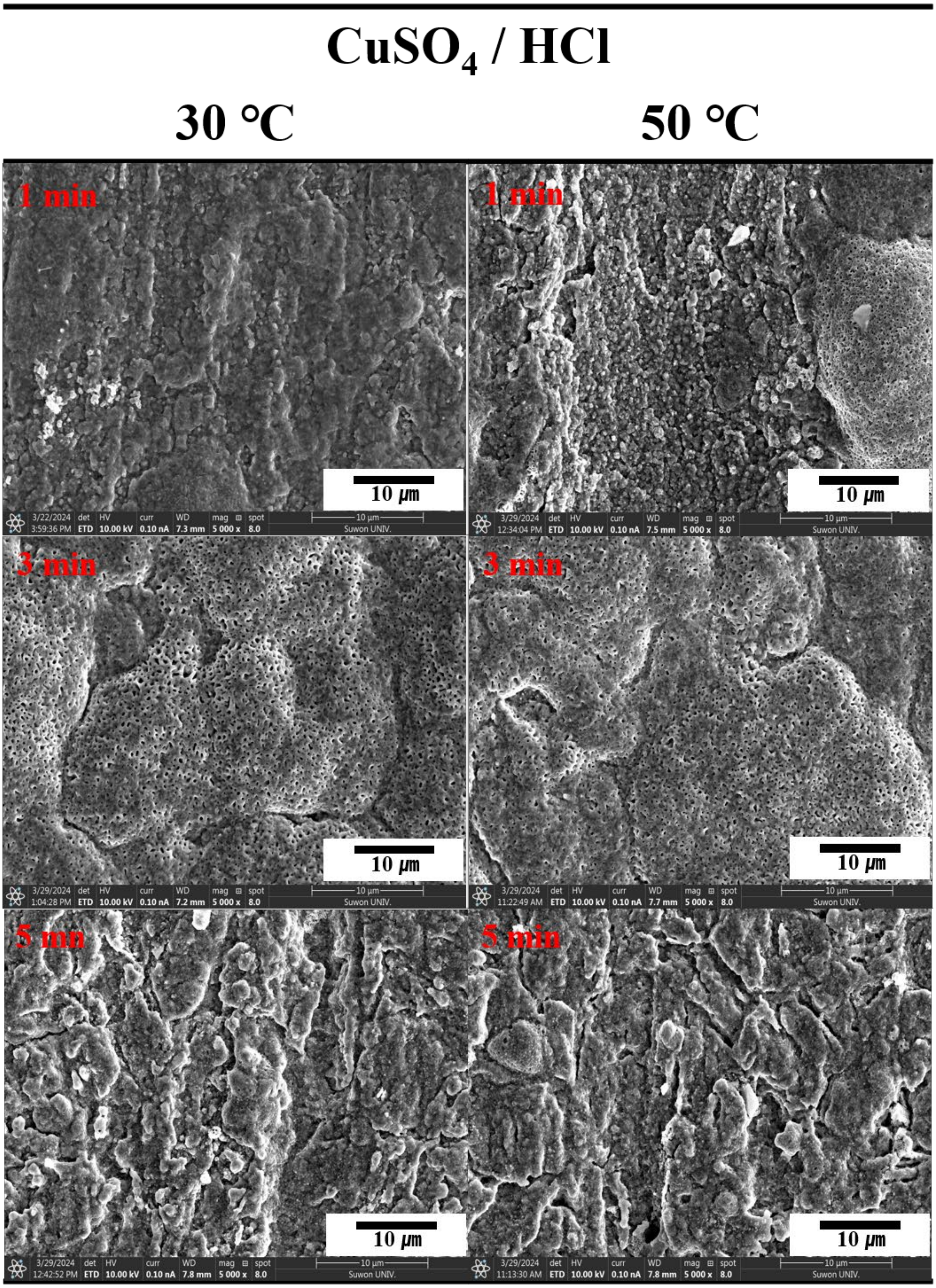

Figure S6. SEM images of SUS surfaces treated by $CuSO_4$/HCl with different etching temperatures and times after annealing.

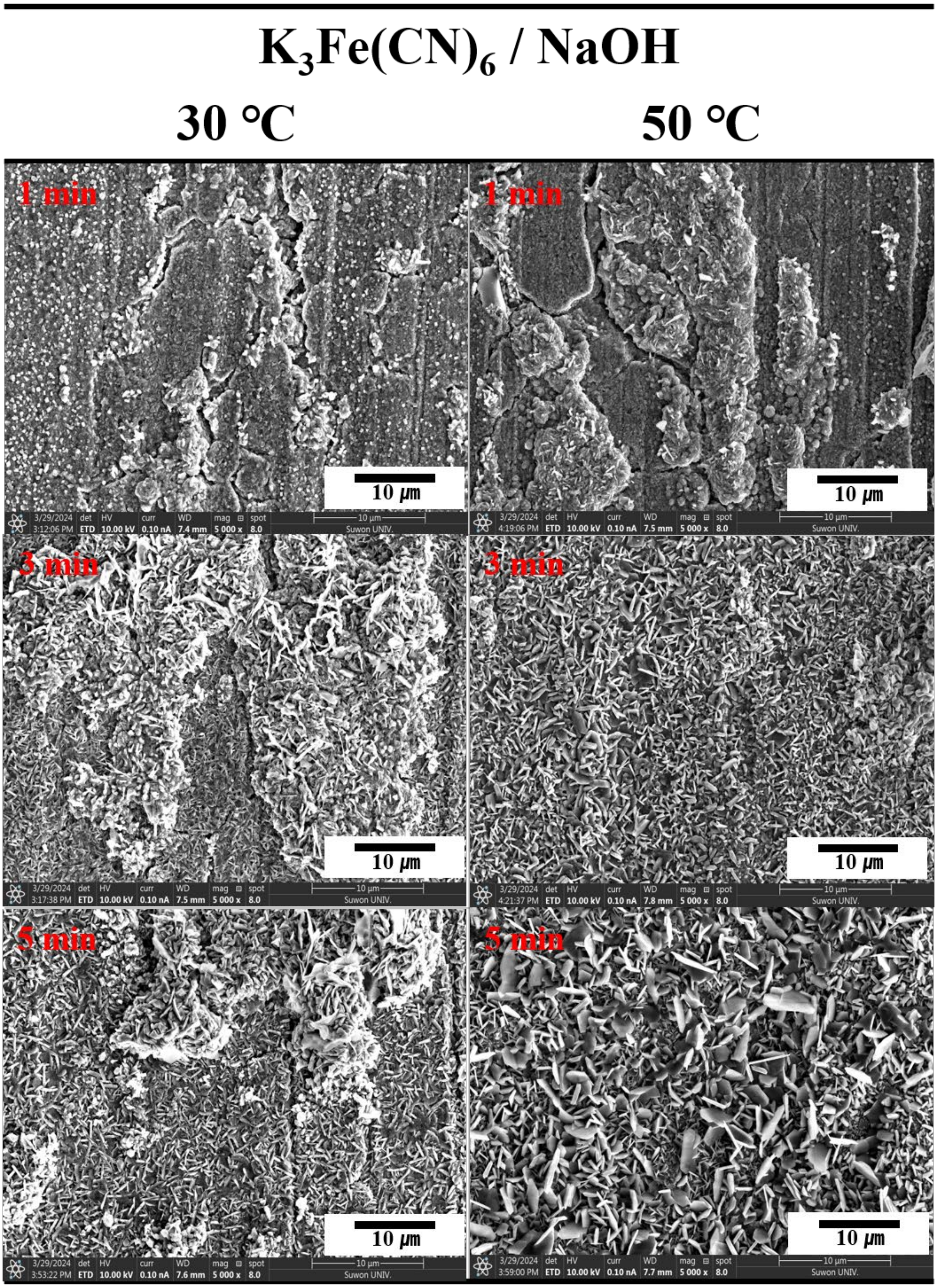

Figure S7. SEM images of SUS surfaces treated by $K_3Fe(CN)_6$/NaOH with different etching temperatures and times after annealing.

HNO_3 / HCl

30 °C | **50 °C**

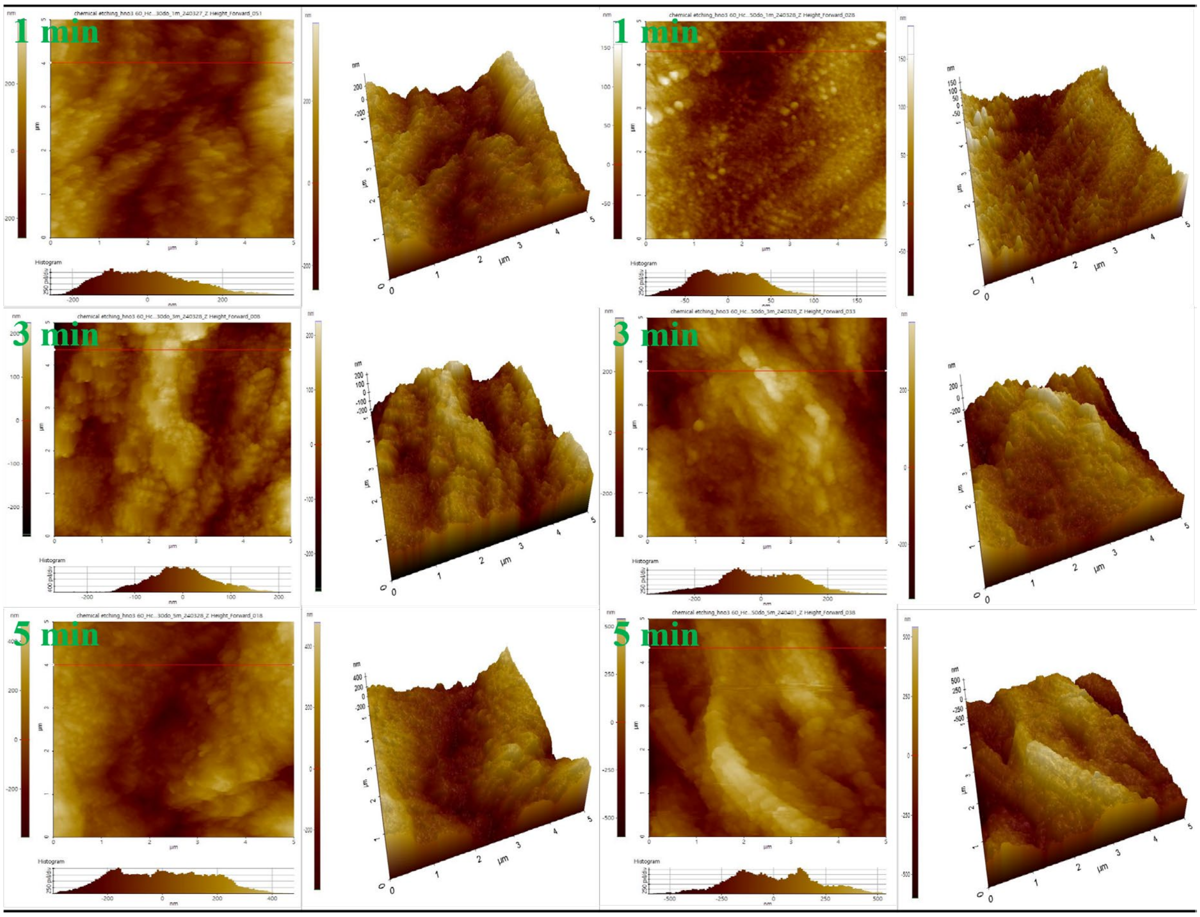

Figure S8. AFM images of SUS treated by HNO_3/HCl with different etching temperatures and times before annealing.

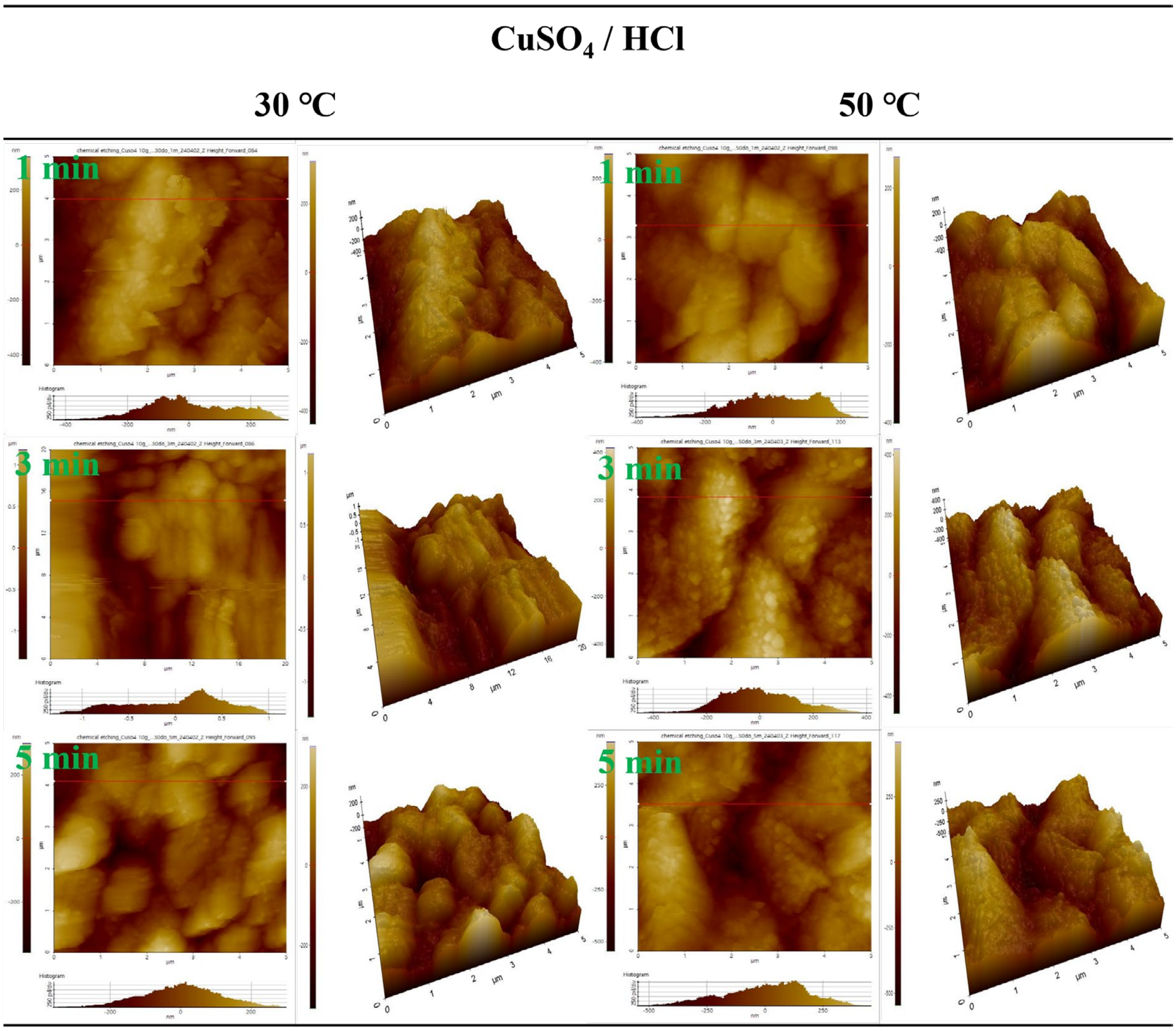

Figure S9. AFM images of SUS treated by $CuSO_4$/HCl with different etching temperatures and times before annealing.

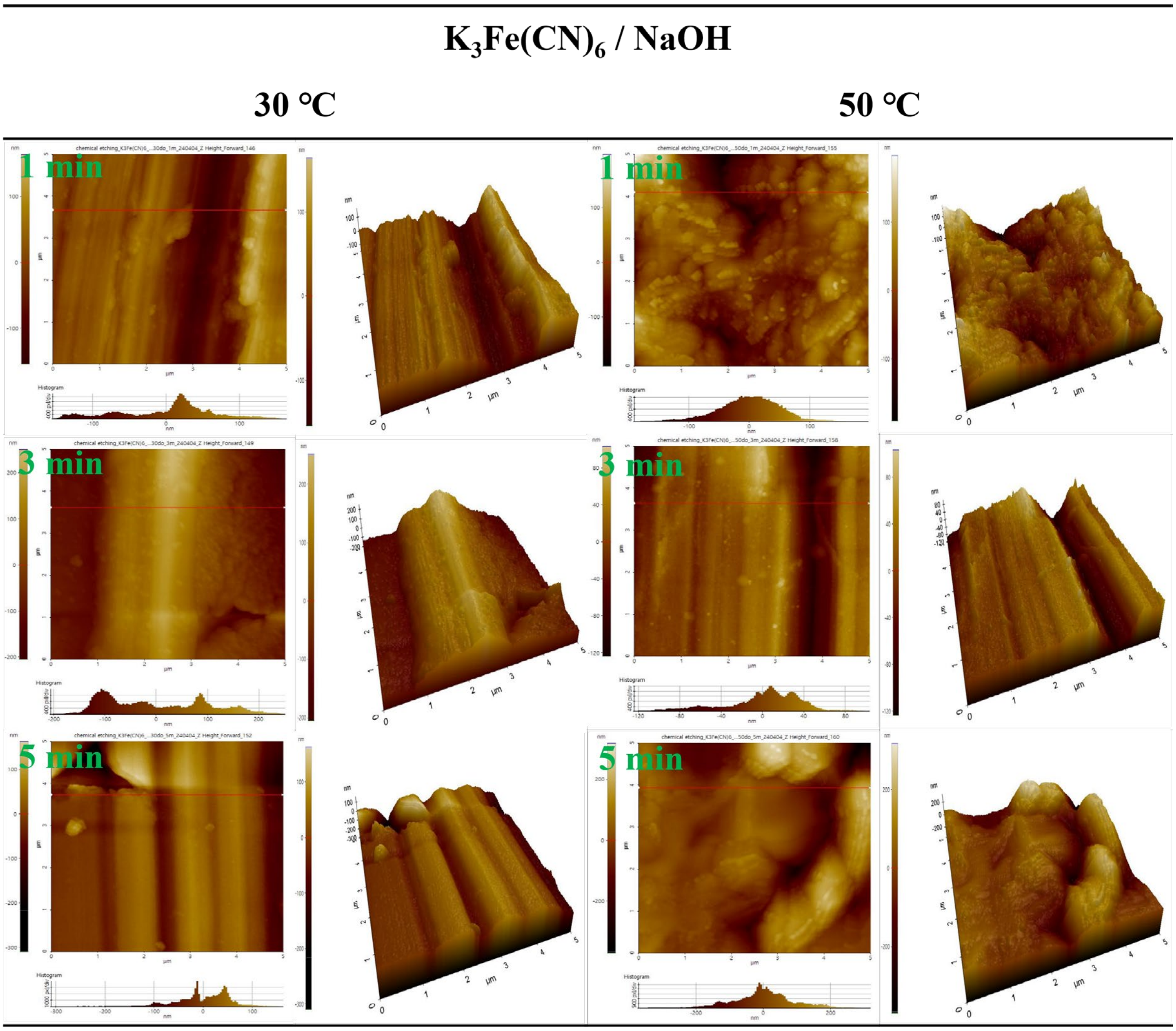

Figure S10. AFM images of SUS treated by $K_3Fe(CN)_6$/NaOH with different etching temperatures and times before annealing.

HNO_3 / HCl

30 °C	50 °C

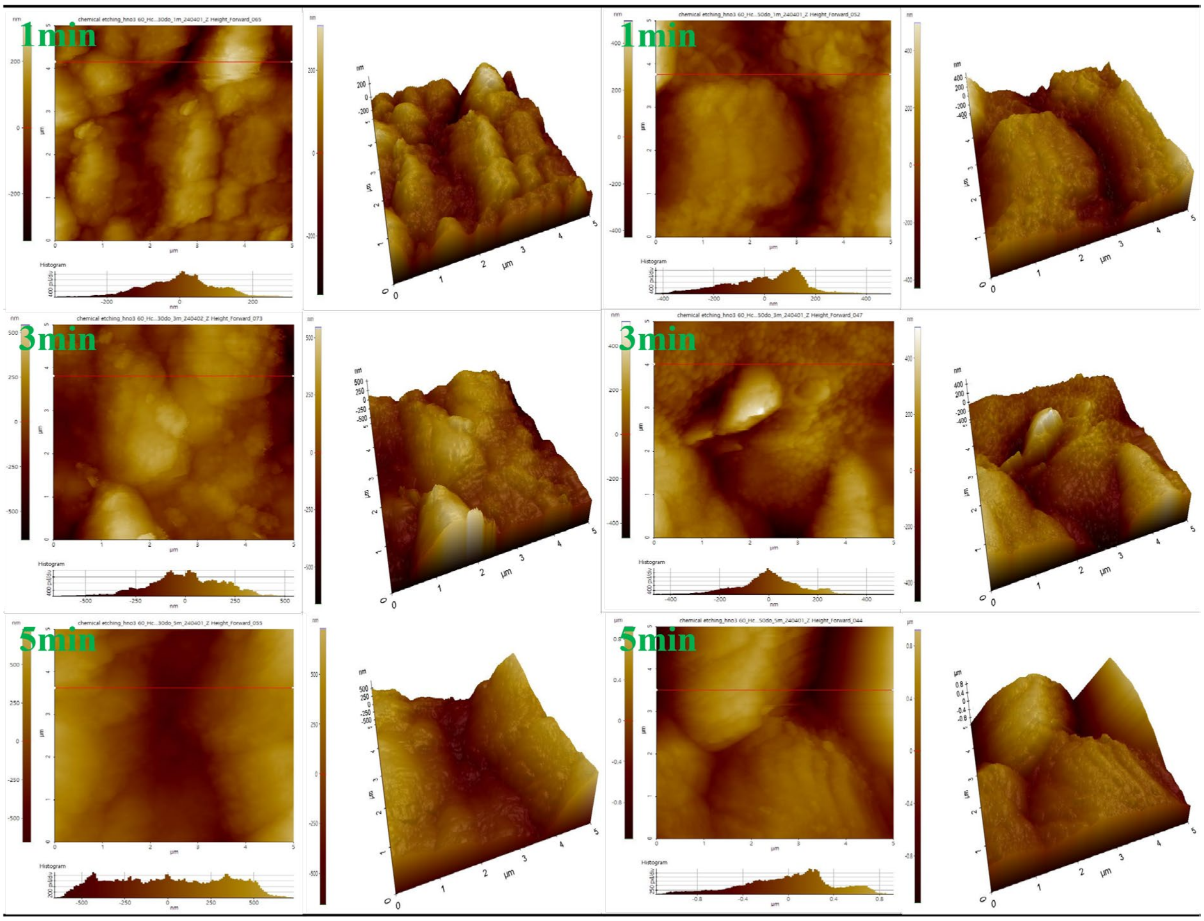

Figure S11. AFM images of SUS treated by HNO_3/HCl with different etching temperatures and times after annealing.

$CuSO_4$ / HCl

30 °C	50 °C

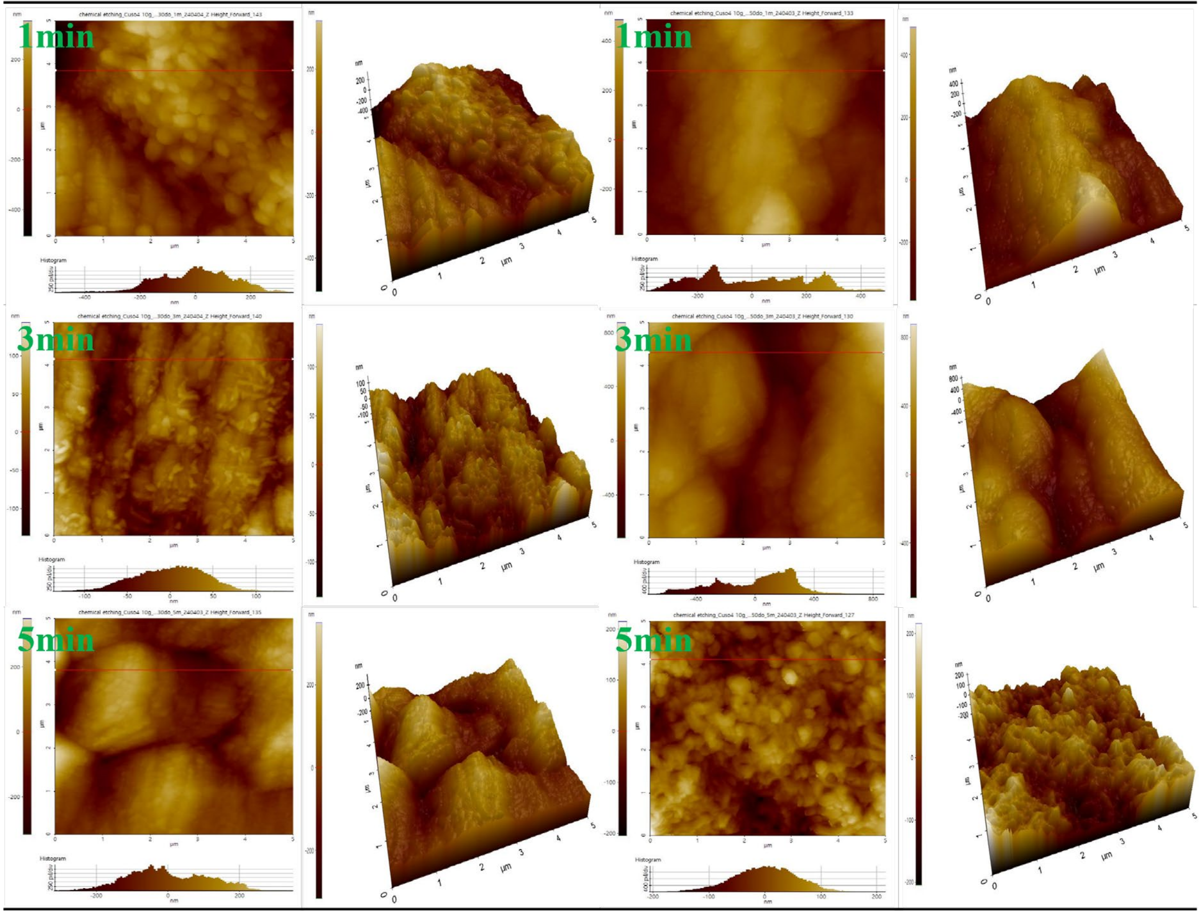

Figure S12. AFM images of SUS treated by $CuSO_4$/HCl with different etching temperatures and times after annealing.

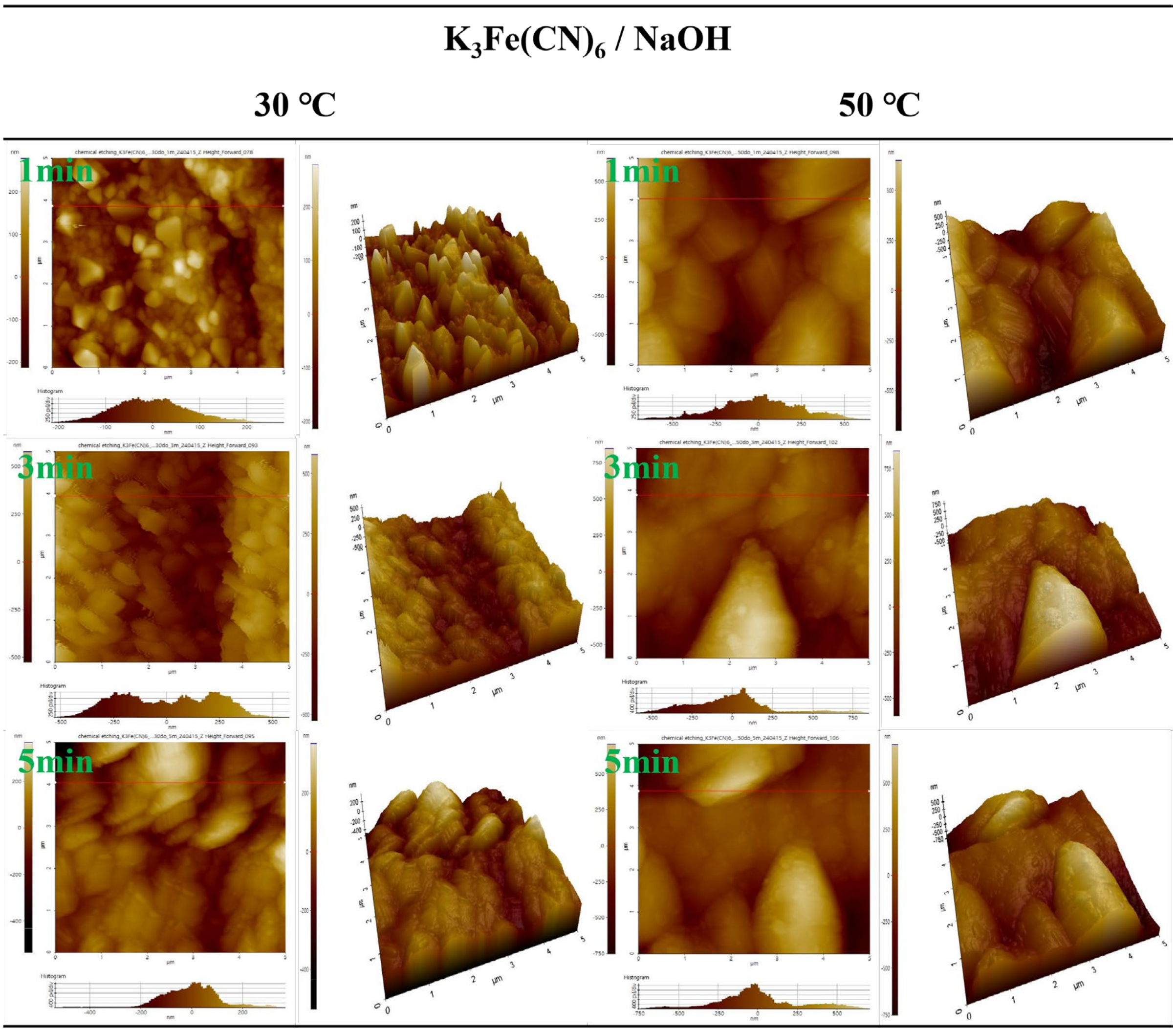

Figure S13. AFM images of SUS treated by $K_3Fe(CN)_6$/NaOH with different etching temperatures and times after annealing.

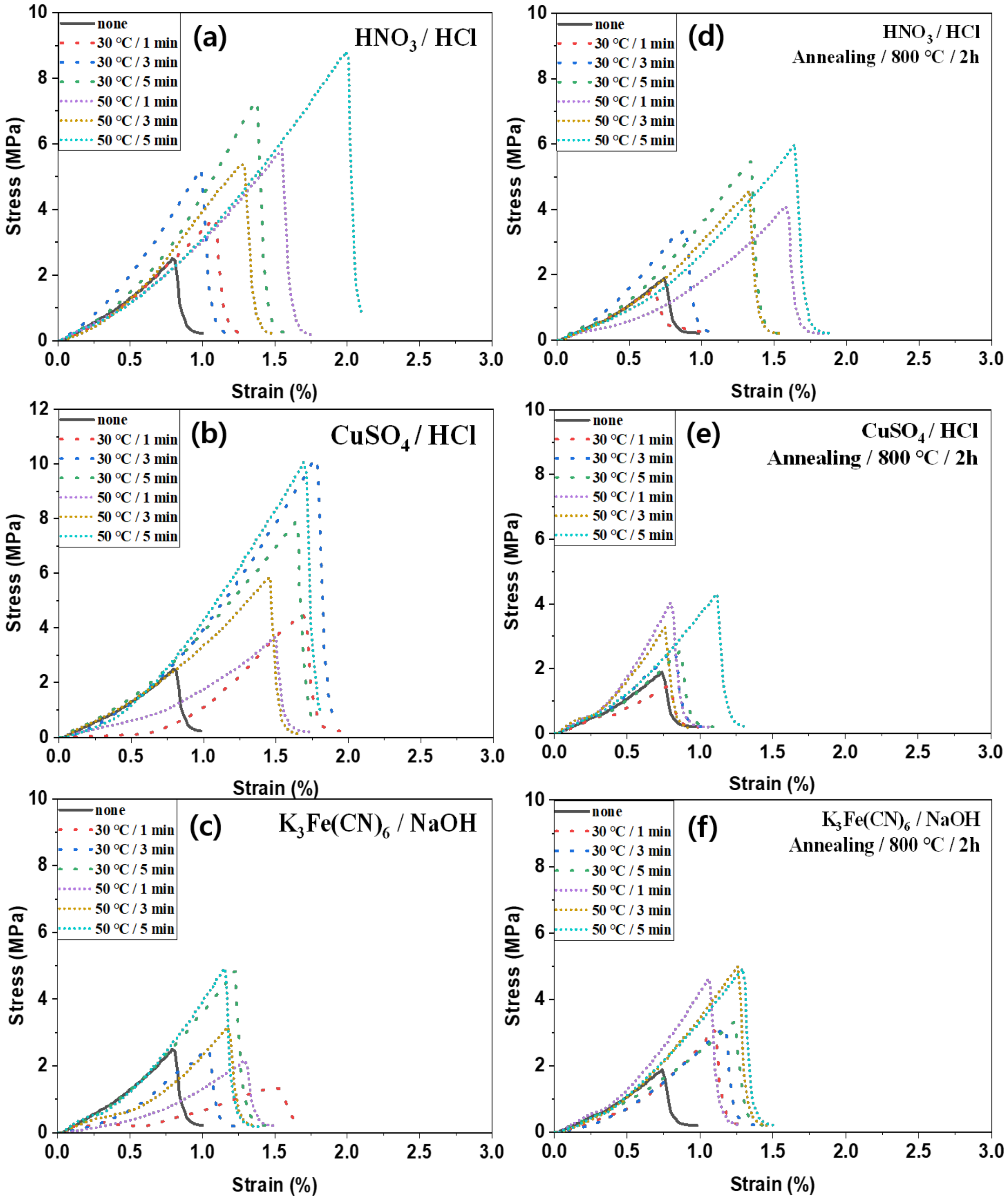

Figure S14. Stress–strain curves of heterojunction bilayer SUS/PA66 composites using SUS with different etching temperatures and times before and after annealing.

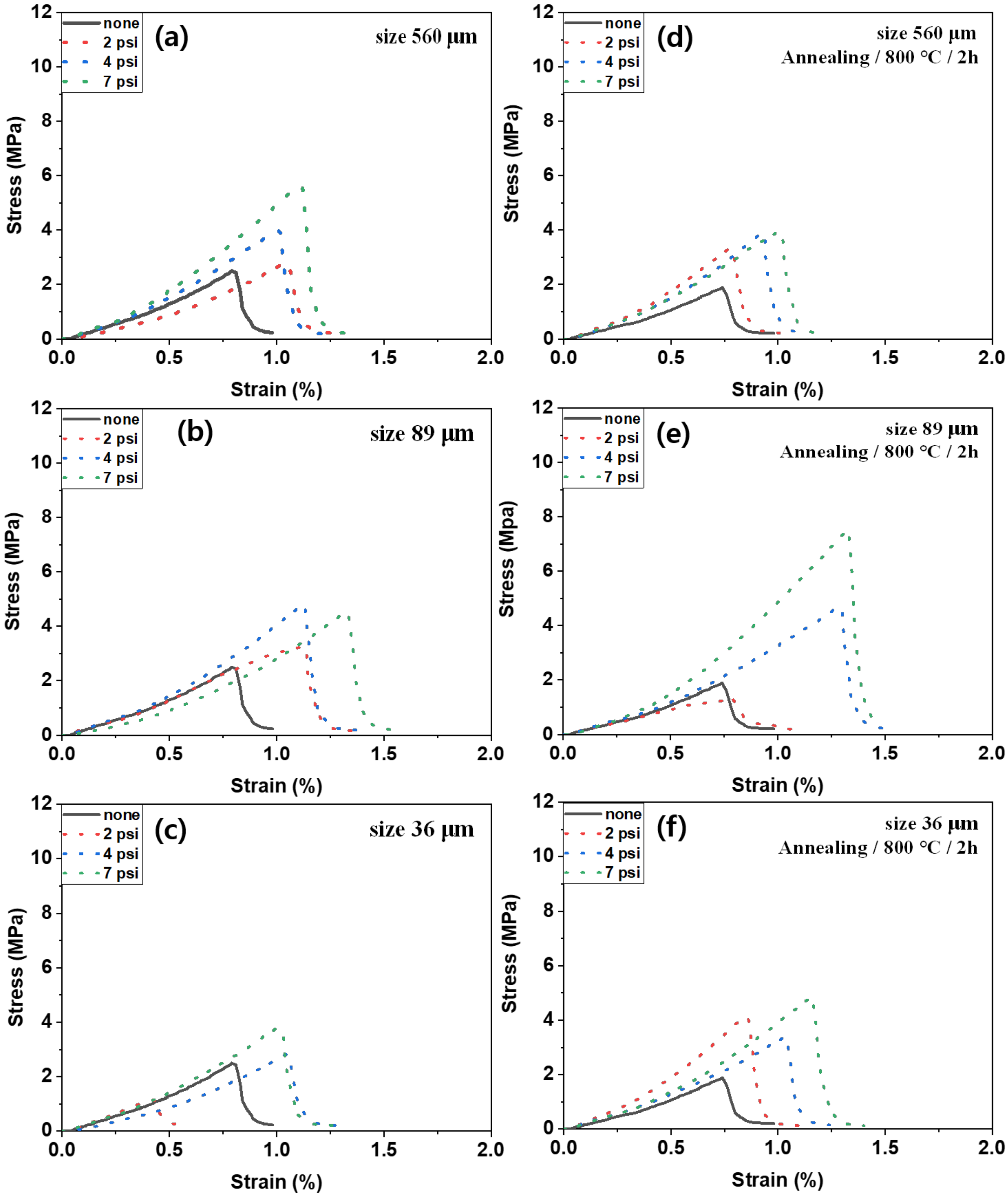

Figure S15. Stress–strain curves of heterojunction bilayer SUS/PA66 composites using SUS with different sandblasting conditions (blasting abrasive size and pressure) before and after annealing.

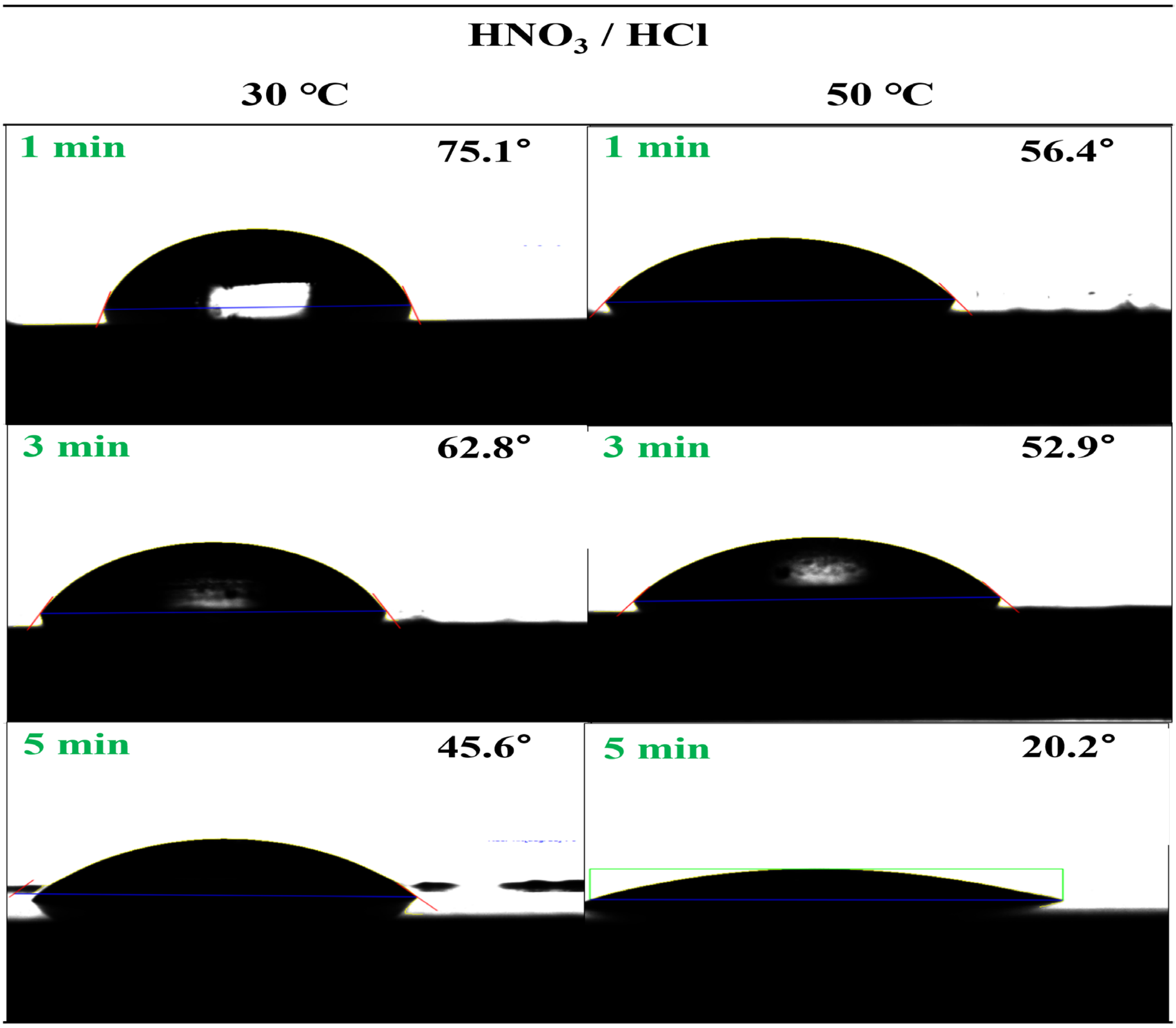

Figure S16. Contact angle images of SUS treated by HNO_3/HCl with different etching temperatures and times before annealing.

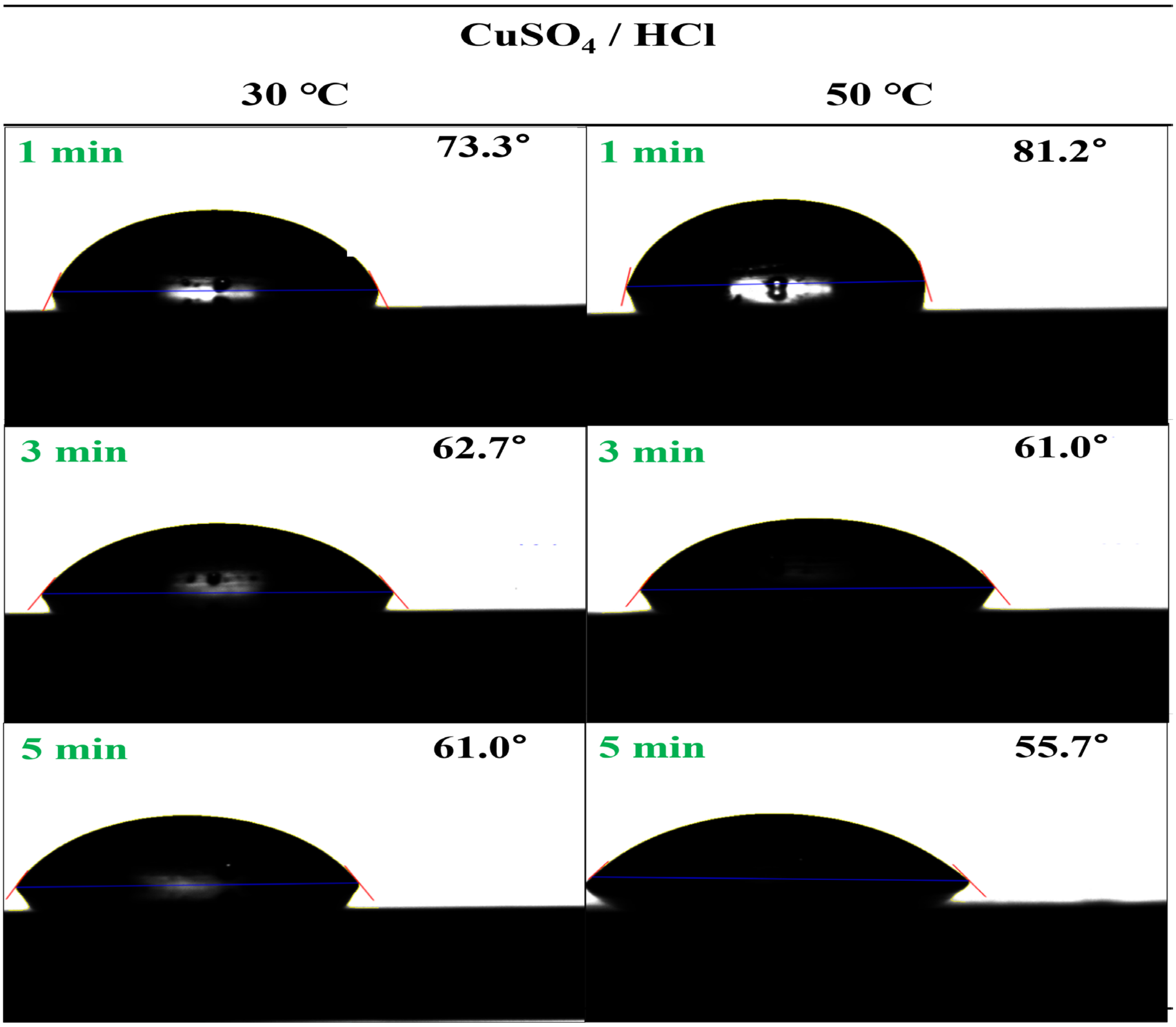

Figure S17. Contact angle images of SUS treated by $CuSO_4$/HCl with different etching temperatures and times before annealing.

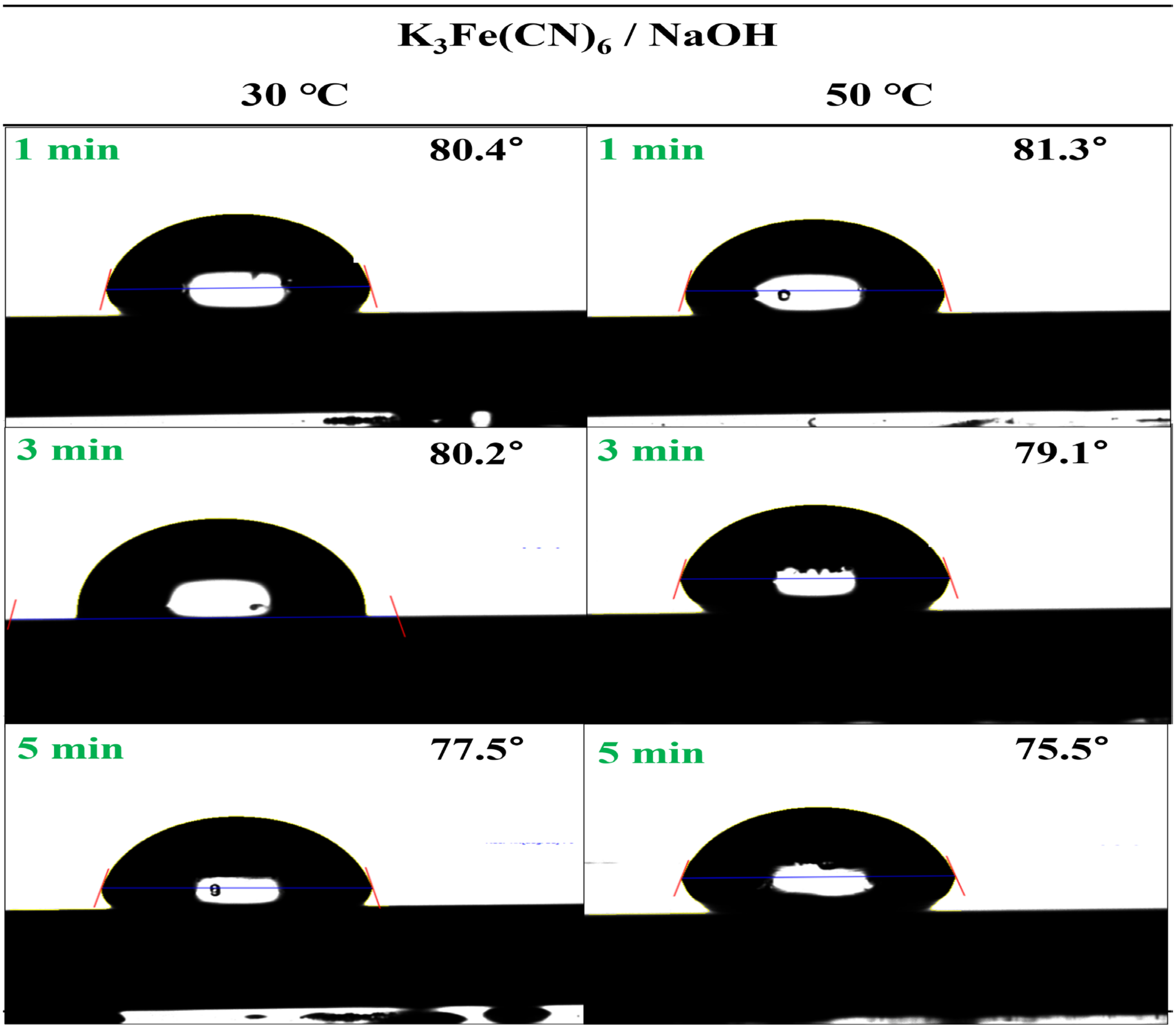

Figure S18. Contact angle images of SUS treated by $K_3Fe(CN)_6$/NaOH with different etching temperatures and times before annealing.

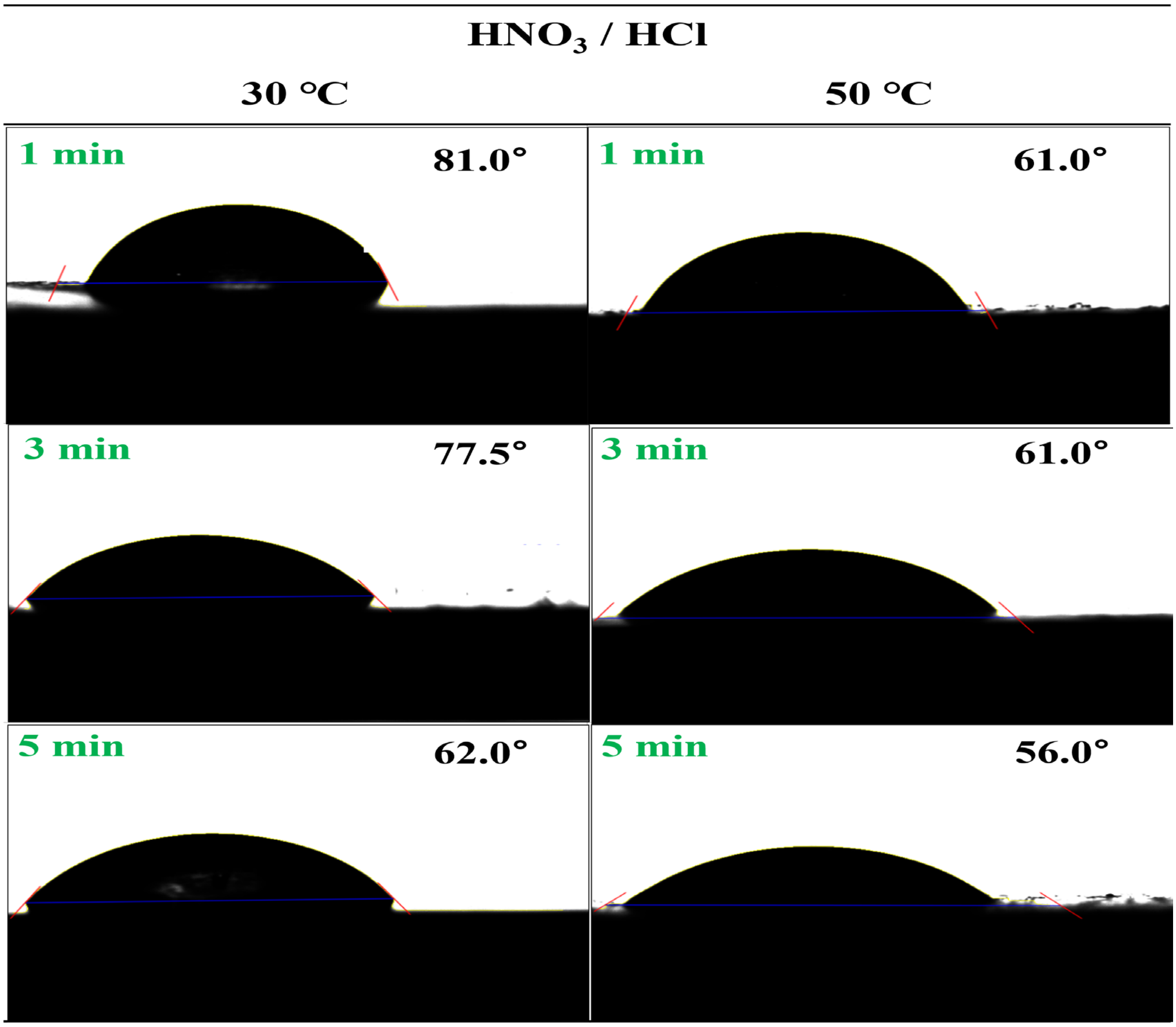

Figure S19. Contact angle images of SUS treated by HNO_3/HCl with different etching temperatures and times after annealing.

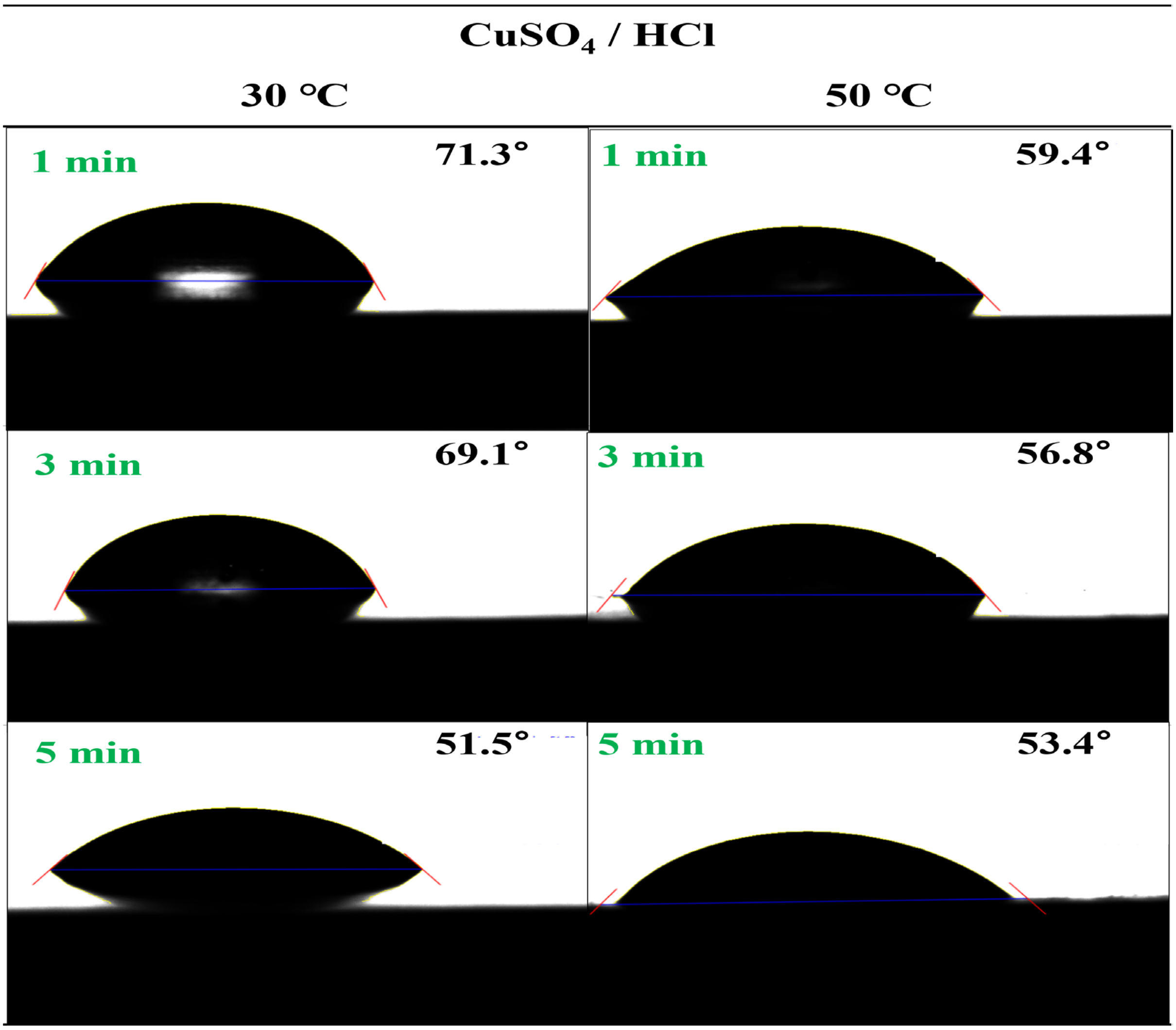

Figure S20. Contact angle images of SUS treated by $CuSO_4$/HCl with different etching temperatures and times after annealing.

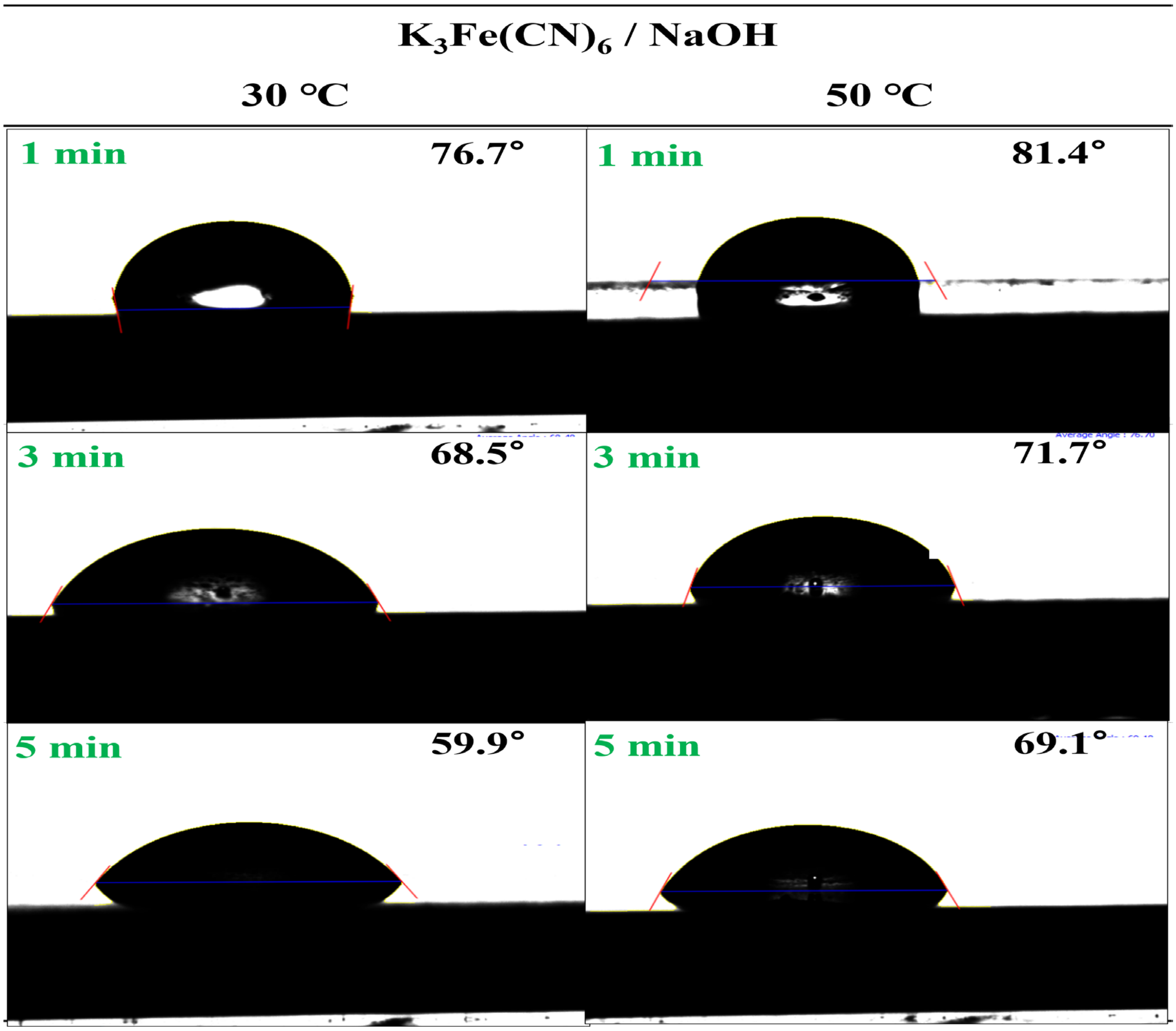

Figure S21. Contact angle images of SUS treated by $K_3Fe(CN)_6$/NaOH with different etching temperatures and times after annealing.

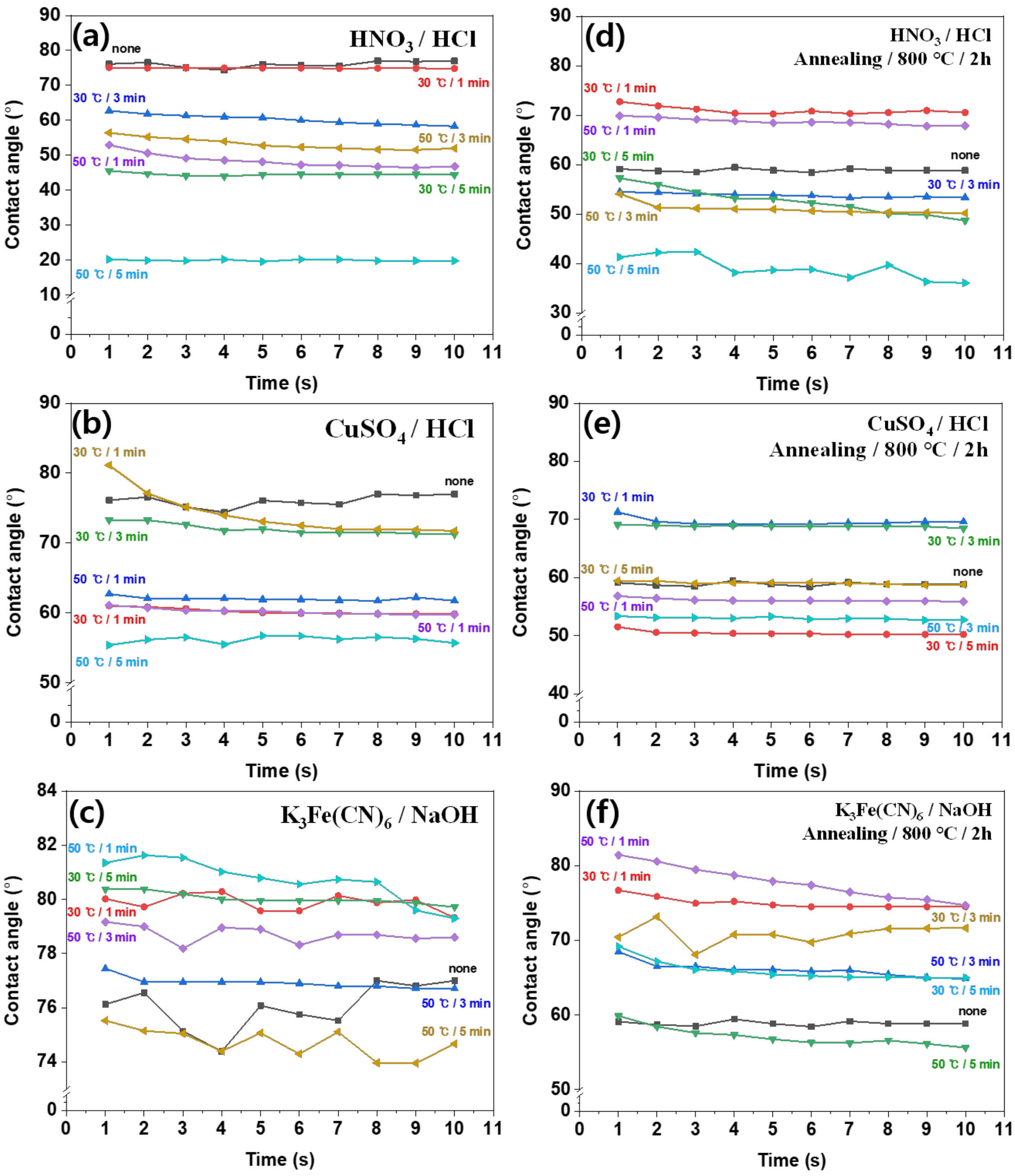

Figure S22. Contact angles of SUS treated with different etching solutions and conditions before (a–c) and after (d–f) annealing: (a) HNO_3/HCl, (b) $CuSO_4$/HCl, (c) $K_3Fe(CN)_6$/NaOH, (d) HNO_3/HCl/annealing, (e) $CuSO_4$/HCl/annealing, and (f) $K_3Fe(CN)_6$/NaOH/annealing.

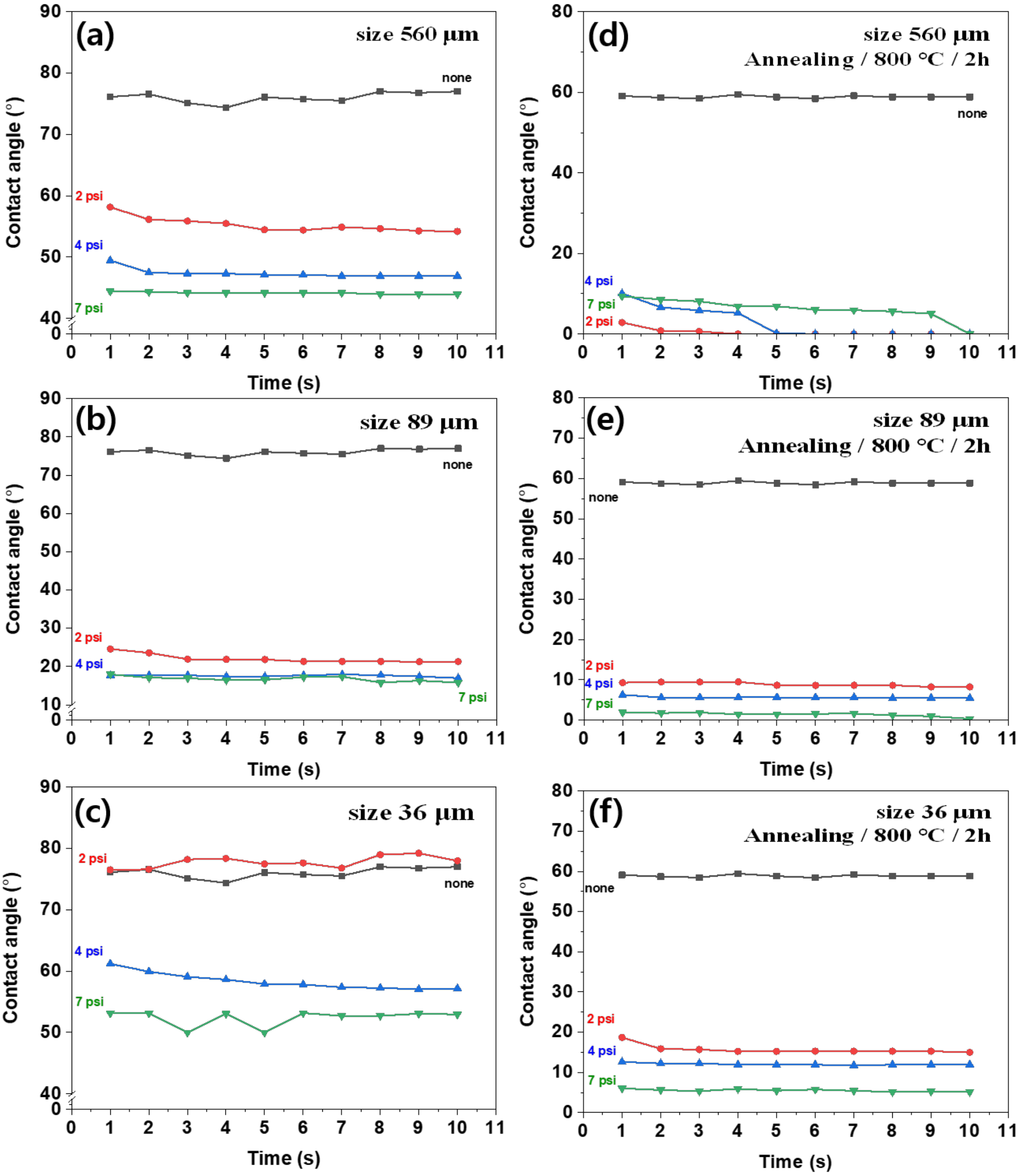

Figure S23. Contact angles of heterojunction bilayer SUS/PA66 composites with different blasting conditions (blasting abrasive size and pressure) before (a–c) and after (d–f) annealing: (a) 560 μm, (b) 89 μm, (c) 36 μm, (d) 560 μm/annealing, (e) 89 μm/annealing, and (f) 36 μm/annealing.
